# Sofosbuvir-Velpatasvir (Epclusa)-Associated Photosensitivity in a Hepatitis C Patient: Case Report and Review of Photosensitivity to Hepatitis C Antiviral Agents

**DOI:** 10.7759/cureus.16496

**Published:** 2021-07-19

**Authors:** Stephen E Glombicki, Philip R Cohen

**Affiliations:** 1 McGovern Medical School, University of Texas Health Science Center, Houston, USA; 2 Dermatology, San Diego Family Dermatology, National City, USA

**Keywords:** antiviral, epclusa, hepatitis, hepatitis c, photoallergic, photosensitivity, phototoxic, simeprevir, sofosbuvir, velpatasavir

## Abstract

Hepatitis C is a ribonucleic acid (RNA) virus, and its presence in individuals was previously considered to be a chronic condition. However, recent therapeutic advances with virus-directed treatment have resulted in a significant increase in cure rates as demonstrated by an absence of virus on repeat testing. Currently, several individual and combination antiviral therapies are available for the treatment of hepatitis C virus infection. However, each of the hepatitis C antiviral agents is associated with potential adverse skin effects whose incidence varies depending on the agent used for treatment. The cutaneous reactions, including photosensitivity, usually resolve once the antiviral treatment is completed. In this report, we discuss the case of a chronic hepatitis C patient who developed sofosbuvir-velpatasvir (Epclusa)-associated toxicity, while receiving the antiviral therapy. The 57-year-old man developed cutaneous phototoxicity when he started treatment with the drug. The adverse skin reaction promptly resolved once the treatment was completed. Clinicians who manage patients being treated with antiviral agents for hepatitis C infection should consider counseling these individuals regarding photoprotection including avoidance of sun exposure, daily sunscreen use, and wearing photoprotective clothing.

## Introduction

Approximately 71 million people in the world live with chronic hepatitis C. In the Western world, chronic hepatitis C virus appears to be the major risk factor for hepatocellular carcinoma. New treatments can cure more than 90% of hepatitis C infections; however, poor access to diagnosis or to care may account for the high prevalence of individuals with the infection [1-4].

Combination drugs for the treatment of hepatitis C have recently been created. Two of the agents currently approved by the Food and Drug Administration are glecaprevir-pibrentasvir (Mavyret) and sofosbuvir-velpatasvir (Epclusa). These medications are generally well tolerated with only mild adverse reactions reported, such as fatigue, headache, insomnia, nasopharyngitis, nausea, and skin rashes [2,5-7].

There are several dermatologic conditions associated with hepatitis C; these include not only photosensitivity, but also leukocytoclastic vasculitis, lichen planus, necrolytic acral erythema, and porphyria cutanea tarda. In addition, changes observed in some patients with hepatitis C may include mixed cryoglobulinemia or elevated porphyrins in the serum or in the urine, or both. Leukocytoclastic vasculitis and elevated porphyrin levels (and porphyria cutanea tarda) have been observed in hepatitis patients with higher viral loads; the elevated porphyrins may, in part, be responsible for hepatitis C-associated photosensitivity. In contrast, necrolytic acral erythema (which can have features of a photosensitivity eruption) has been associated with lower hepatitis C viral loads; this brings the possibility that the photosensitivity observed during hepatitis treatment may be related to either the effect of the therapy resulting in lowering the hepatitis C viral load (and thereby resulting in photosensitivity similar to necrolytic acral erythema) or the single drug or the combination of agents being photosensitizers or both [1-7].

Cutaneous photosensitivity may be drug-associated and includes photoallergic and phototoxic reactions to the medication. Photoallergic reactions are uncommon, require prior exposure to the drug, can occur with low doses of the drug, typically do not manifest after the initial exposure, develop one to three days after initiation of the drug, present with morphology similar to dermatitis, originate on sun-exposed sites, and subsequently evolve to potentially include the entire body with the appearance of eczematous dermatitis. In contrast, phototoxic reactions are more common, do not require prior exposure to the medication, increase in frequency with higher doses of the drug, may occur after the initial treatment, often begin within minutes to hours after receiving the drug, present with erythematous and edematous lesions, and may even include blisters that morphologically mimic an acute sunburn whose distribution is restricted to areas exposed to the sun [8,9].

Albeit uncommon, a drug-associated rash may be associated with sofosbuvir-velpatasvir. We describe the case of a man with hepatitis C who developed cutaneous phototoxicity while undergoing this antiviral treatment. In addition, we engage in a review of drug-induced photosensitivity that has been associated with other antiviral agents used for treating hepatitis C [1-3,5,10-17].

## Case presentation

A 57-year-old man presented for the evaluation of a new rash on his face and arms. He had a prior dermatologic history of skin cancer. A squamous cell carcinoma on his left lower leg had been excised without recurrence a year prior. His past medical history was remarkable for essential hypertension, fatty liver, gallbladder polyp, gastritis, hiatal hernia, major depressive disorder, osteoarthritis of the knees, right kidney stone, post-traumatic stress disorder, and severe gastroesophageal reflux disease. He also smoked half a pack of cigarettes each day. His daily medications included mirtazapine, omeprazole, polyethylene glycol 3350, prazosin, venlafaxine, and vitamin D3.

In addition, he had chronic hepatitis C (genotype three). He had acquired the infection 35 years ago, during the summer of 1986, secondary to three episodes of intravenous drug use with his then-girlfriend. In the late 1990s, his hepatitis C infection had been diagnosed by liver biopsy; however, there had been no available treatment options at that time.

An ultrasound of his liver showed its size to be at the upper limit of normal, with significant fibrosis [stage Fibrosis two (F2), with normal being Fibrosis zero (F0) or Fibrosis one (F1) and cirrhosis being Fibrosis four (F4)]. He was hepatitis B-immune and his alpha-fetoprotein tumor marker level was 1.9 ng/mL, which is within the normal limit (0-8 ng/mL) for adults. His hepatitis C viral load was 927,000 IU/L (low viral load is defined as less than 800,000 IU/L).

He had started antiviral therapy 2.5 months ago. He was being treated with a daily oral combination dose of 400 mg sofosbuvir and 100 mg velpatasvir (Epclusa). Three weeks into his 12-week course of sofosbuvir-velpatasvir, his hepatitis C RNA was not detected on polymerase chain reaction. He presented to the dermatology office for examination with three weeks remaining of his 12-week course. He reported that the onset of his skin rash occurred shortly after the initiation of his antiviral treatment.

**Figure 1:**
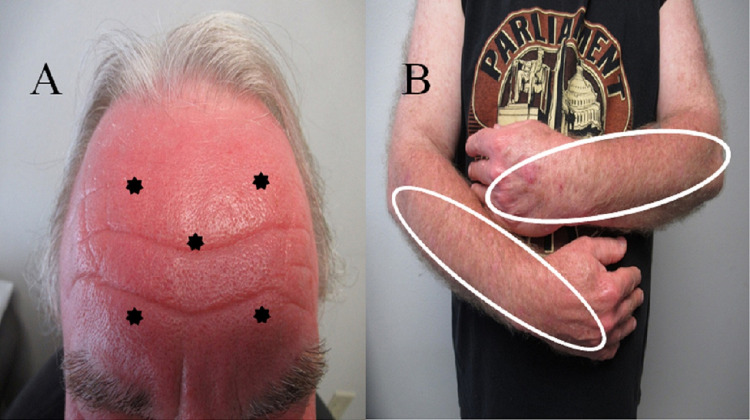
Clinical presentation of sofosbuvir-velpatasvir (Epclusa)-associated photosensitivity The forehead (A) and the upper extremities (B) of a 57-year-old man who developed a phototoxic skin eruption after beginning antiviral therapy for his chronic hepatitis C virus infection with sofosbuvir-velpatasvir (Epclusa). There is confluent erythema not only on his forehead (black stars) but also on his distal arms, forearms, and dorsal hands (within the white circles).

Cutaneous examination showed prominent confluent erythema distributed in areas that had sun exposure (Figure 1). Specifically, his entire face was red. In addition, the extensor surfaces of his distal arms (from just above the elbows), forearms, and dorsal hands also had confluent erythema and hyperpigmentation; these were also locations that had been exposed to the sun. The unaffected areas on his proximal arms corresponded to the skin area that was usually covered by his shirt.

The correlation of his clinical history with the morphology and the distribution of his skin eruption established a diagnosis of photosensitivity to sofosbuvir-velpatasvir. Sunscreen and photoprotective clothing (long-sleeve shirts, gloves, and a hat) were recommended. He completed his antiviral therapy during the next three weeks. Subsequent follow-up, two months after discontinuing sofosbuvir-velpatasvir, showed complete resolution of the phototoxic skin rash.

## Discussion

Hepatitis C virus infection may present as either acute infection or chronic disease without an initial inflammatory phase. Although jaundice may be the most obvious and ominous clinical sign, patients may present with non-specific symptoms such as anorexia, arthralgia, choluria, fatigue, fever, malaise, myalgia, nausea, vomiting, and weight loss. In addition, liver enzymes (such as alanine aminotransferase and aspartate transaminase) may be elevated [1,18].

Mechanisms of hepatitis C viral transmission include sharing syringes with intravenous drug users and sexual contact without barrier contraception. In the 20th century, prior to when viral RNA screening tests in blood became available, blood transfusions and organ donation were also common routes of transmission. Another mechanism of transmission is occupational exposure. The incidence of seroconversion from a contaminated needle in the healthcare field is estimated to be 1.8% [1,2].

Spontaneous elimination of the hepatitis C virus may occur in some patients. However, in a high proportion of infected individuals, it will evolve into a chronic form of hepatitis C virus, defined as persistence of viral RNA for more than six months after the exposure to the virus, with the possibility of complications such as liver fibrosis that progresses to cirrhosis and hepatocellular carcinoma. Serologic (hepatitis C virus antibody) and confirmatory molecular tests (hepatitis C virus RNA) are needed to diagnose chronic hepatitis C [1,19].

Prior to initiating treatment, additional evaluation is necessary to determine hepatitis C virus genotype and the extent of liver fibrosis. Risk factors can modify the history of disease progression and accelerate the fibrotic process. These include advanced age, alcohol use, male sex, virus genotype three, and tobacco use [1,4].

The hepatitis C virus is round in shape. It is a small, single-stranded, positive-strand, enveloped RNA virus. It belongs to the *Hepacivirus* genus within the *Flaviviridae* family [1,11]. The genome of the hepatitis C virus is 9600 base pairs long and is stored in an icosahedral capsid. Several major genotypes exist, and genotypes one to three have a worldwide distribution. The virus encodes a unique polyprotein that is processed while being translated into several proteins [1,3,19].

A nonstructural (NS) protein is a protein encoded by a virus but not part of the viral particle. For hepatitis C virus, examples include NS3, NS4A, NS4B, NS5A, and NS5B. The genome acts as messenger RNA for the host cell translational machinery to synthesize the polyprotein, which then gets broken up by proteases to generate different parts of the virus. These proteins are the target for second-generation direct-acting antiviral treatment [1-4,20].

Current antiviral therapy for hepatitis C includes several individual medications or combination agents consisting of two drugs (Table 1) [1-4]. NS5A inhibitors, such as velpatasvir and ledipasvir, inhibit NS5A, a viral phosphoprotein that plays a key role in RNA replication. NS5B inhibitors, such as sofosbuvir, inhibit NS5B, an RNA-dependent RNA polymerase acting as a chain terminator, which ultimately prevents viral RNA replication. NS3/4A inhibitors, such as grazoprevir, simeprevir, and voxilaprevir, inhibit NS3/4A, a viral protease, which prevents viral replication. Ribavirin is an alternative drug that inhibits the synthesis of guanine nucleotides by competitively inhibiting inosine monophosphate dehydrogenase, thus impairing viral RNA and deoxyribonucleic acid (DNA) replication. It is used as an adjunct for patients whose hepatitis C infection is refractory to newer medications; however, it has been associated with an increased incidence of pruritus and rash [3].

**Table 1:** Common suffixes of different hepatitis C antiviral medications* *Each category of hepatitis C antiviral medications in this table affects or interferes with a nonstructural protein that is essential for hepatitis C virus replication ^a^The "a" of "-asvir" corresponds with the "A" of "NS5A" ^b^The "b" of "-buvir" corresponds with the "B" of "NS5B" ^c^The "pr" of "-previr" corresponds with the "pr" of "protease inhibitor" HCV: hepatitis C virus; NS: nonstructural protein; RNA: ribonucleic acid

Suffixes of hepatitis C antiviral medications and mechanism of action			
Suffix	-asvir^a^	-buvir^b^	-previr^c^
Mechanism of action	Inhibits NS5A (viral phosphoprotein)	Inhibits NS5B (nucleotide analog RNA-dependent RNA polymerase)	Inhibits NS3/4A (HCV protease)
Reference	[1-4]	[1-4]	[1-4]

The patient in this report was treated with sofosbuvir-velpatasvir. The agent was approved by the Food and Drug Administration in 2016. The treatment consists of daily oral administration of the agent for 12 weeks; this is a much shorter course of therapy compared to the old standard of 48 weeks of pegylated interferon alfa and ribavirin [6,7].

In clinical trials involving the single drug sofosbuvir, there were no clinically significant adverse effects beyond anemia, dizziness, fatigue, headache, insomnia, nausea, and pruritus. In addition, studies in rats and rabbits suggest that velpatasvir is not phototoxic. Indeed, the most common adverse events in patients taking the combination antiviral agent, sofosbuvir-velpatasvir, were fatigue, headache, insomnia, nasopharyngitis, and nausea. Investigators have rarely observed cutaneous reactions to the agent; only 2% of patients receiving sofosbuvir-velpatasvir experienced a rash associated with the drug [2,6,7].

All of the other antiviral agents that are used to treat hepatitis C also have potential systemic adverse events or cutaneous side effects or both. In particular, the drug-associated skin reactions for many of these agents are only described as a rash. However, some of these patients experienced photosensitivity while taking these medications: (Table 2) [1,2,3,5,14,15,17] and (Table 3) [2,10-13,16].

**Table 2:** Photosensitivity to combination hepatitis C antiviral agents, consisting of only nonstructural protein inhibitors ^a^Case reports of photosensitivity were not able to be retrieved using the PubMed search engine ^b^When given alone, the incidence of rash or pruritus was 0%. When given with ribavirin, moderate or severe rash or pruritus incidence was 4%. Post-marketing cases of angioedema reported ^c^Pruritus was noted in 17% of patients with severe renal impairment. Pruritus was also noted in 7% of patients with liver or kidney transplants. Rash was noted in 4% of pediatric patients (3-12 years old); one patient had to discontinue the drug because of a grade three erythematous rash. Post-marketing cases of angioedema reported ^d^Agent discontinued in 2018 ^e^Photosensitivity to sofosbuvir alone was not observed; however, triple-drug therapy was a failed experimental combination ^f^No dermatologic adverse effects reported on the package insert ^g^All photosensitivity reactions (18 of 40 patients) were related to simeprevir; however, in five of these patients, the reaction was also considered to be related to ledipasvir-sofosbuvir ^h^Mild or moderate rash was observed in 2% of patients. In patients on Epclusa and ribavirin, rashes were observed in 5%. Post-marketing cases of angioedema and skin rashes reported, sometimes with blisters or angioedema-like swelling 3/4AI: NS3/4A inhibitor; 5AI: NS5A inhibitor; 5BI: NS5B inhibitor; CR: current report; NS: nonstructural protein; Photo: incidence of photosensitivity; Ref: reference; SP: single patient; TP: two patients

Combination agents	Mechanism of action	Photo	Ref
Elbasvir-grazoprevir (Zepatier)^a, b^	5AI - 3/4AI	0%	[1]
Glecaprevir-pibrentasvir (Mavyret)^a, c^	3/4AI - 5AI	0%	[5]
Ledipasvir, simeprevir^d^, and sofosbuvir^e^	5AI, 3/4AI, and 5BI	45%	[14]
Ledipasvir-sofosbuvir (Harvoni)^f^	5AI - 5BI	12.5%^g^, SP	[14,15]
Simeprevir^d^ and sofosbuvir	3/4AI and 5BI	5 - 7%, TP	[2,3,17]
Sofosbuvir-velpatasvir (Epclusa)^a, h^	5BI - 5AI	SP	CR

**Table 3:** Photosensitivity to individual or combination hepatitis C antiviral agents with various mechanisms of action ^a^An experimental drug that never received approval ^b^Some brand names have been discontinued (by Merck), such as the oral solution of Rebetol in 2019, for business reasons and not due to any safety, efficacy, or quality issues ^c^A failed experimental combination ^d^Darkened coloration of exposed skin, dermatitis, paresthesia, and pruritic nodules were noted ^e^Seven of 12 patients taking 0.05 mg/kg had photosensitivity [11] ^f^All seven patients taking 0.1 mg/kg had photosensitivity, which resulted in the early discontinuation of the trial [11] ^g^Roferon-A was discontinued from the market in 2007 and Infergen in 2013. These forms of interferon-alpha were replaced by pegylated interferon, which has been further replaced by second-generation direct-acting antivirals ^h^Agent discontinued in 2018 ^i^Some brand names of pegylated interferon alfa-2b have been discontinued, such as Sylatron in 2019. However, Pegasys and PegIntron are still available; yet, due to their poor safety profile, they are not commonly used +: with; +/-: with or without; 3/4AI: NS3/4A inhibitor; 5BI: NS5B inhibitor; DHI: dehydrogenase inhibitor; IC: immunomodulatory cytokine; IMP: inosine monophosphate; NS: nonstructural protein; Photo: incidence of photosensitivity; Ref: reference

Individual or combination agents	Mechanism of action	Photo	Ref
Deleobuvir^a^ and faldaprevir^a^ with or without ribavirin^b^	5BI, 3/4AI +/- IMP DHI	25 - 33%	[10]
Hypericin^c, d^ (derivative of St. John’s wort plant)	Heme oxygenase-1 de-acetylator and down-regulator	58%^e^, 100%^f^	[11,12]
Interferon-alpha^g^ and ribavirin^b^	IC and IMP DHI	20%	[13]
Simeprevir^h^ with both pegylated interferon-alpha^i^ and ribavirin^b^	3/4AI + IC + IMP DHI	1.6 - 4%	[2,16]

Simeprevir is a photodynamically active sulfonamide. Simeprevir’s action spectrum is ultraviolet B (UVB, 290-320 nanometers) and ultraviolet A (UVA, 320-400 nanometers). Thus, absorption of ultraviolet light can lead to adverse effects [2,20]. Simeprevir photosensitivity reactions typically begin between two to eight weeks after starting the treatment and occur more commonly in the summer. They usually appear in body areas that were exposed to the sun, such as the face, neck, upper chest in a V-distribution, and dorsal aspect of the forearms and hands. Mild pruritus has also been often reported [20].

Photosensitivity reactions induced by simeprevir have been dose-dependent, and they even occurred in patients who were using sun-protective measures. In some of the individuals, the drug-induced photosensitivity led to treatment cessation. Indeed, in one study, two patients experienced severe photosensitivity reactions that required hospitalization [2].

A trial that included a combination of simeprevir and sofosbuvir not only demonstrated photosensitivity in 7% of all patients but also an increased risk of photosensitivity or rash among East Asian patients [3]. In addition, photosensitivity developed in 45% (18 of 40 patients) of individuals receiving ledipasvir, simeprevir, and sofosbuvir [14]. In another report, after treatment with simeprevir and sofosbuvir, two patients, a 50-year-old man and a 59-year-old woman, developed a photo-distributed lichenoid eruption; within two weeks after stopping the drugs, the cutaneous eruption completely resolved without recurrence [17]. In 2018, simeprevir was discontinued in the United States [1].

In a study that included 40 individuals who received ledipasvir, simeprevir, and sofosbuvir, 12.5% (five of 40 patients) developed photosensitivity that was thought to be associated with the ledipasvir and sofosbuvir [14]. Similarly, in another report, five weeks after starting ledipasvir-sofosbuvir, a 70-year-old man with post-transfusion hepatitis C (genotype 1b) but without cirrhosis developed well-demarcated mild scaly erythematous plaques on areas that had been exposed to the sun; in addition, scattered flaccid bullae were present on his forearms. His skin lesions not only persisted but also deteriorated after he completed 12 weeks of antiviral agent treatment. The skin over the retroauricular folds, submental area, flexural forearms, and other regions that tend to be covered by clothing, a facial mask, or watch were relatively unaffected [15].

Rash and photosensitivity in patients treated with faldaprevir were dose-dependent. In addition, dermatological adverse effects were more frequently reported in patients receiving the older hepatitis C virus protease inhibitors such as boceprevir, faldaprevir, and telaprevir as part of triple combination regimens. Subsequently, boceprevir and telaprevir are no longer available in the United States, and faldaprevir, which was an experimental agent, never received approval for use in the treatment of hepatitis C [18].

While some of the older hepatitis C protease inhibitors have been associated with severe rashes, this was not observed in trials for the combination of glecaprevir and pibrentasvir (Mavyret) [5]. Also, photosensitivity was not reported with any of the newer agents including sofosbuvir and velpatasvir - either alone or in combination - in not only large series but also phase II and phase III clinical trials [2,5-7].

The treatment of hepatitis C typically involves 12 weeks of therapy. During this period, patients should be counseled to use appropriate photoprotection to prevent drug-associated photosensitivity. This intervention may include not only topical sunscreen but also appropriate clothing (such as hats, long-sleeve shirts, long pants, socks, and possibly even gloves) that prevents exposure of their skin to the sun. However, despite appropriate exposure-limiting modalities, there is still a minimal risk of developing a cutaneous photosensitivity reaction when a patient with hepatitis C is being treated with antiviral therapy.

Our patient had a history of extensive sun exposure; however, he did not develop photosensitivity until after initiating treatment with sofosbuvir-velpatasvir (Epclusa). Similar to our patient, we assume that the photosensitivity observed in hepatitis C patients during hepatitis antiviral treatment is caused by the therapy they receive. However, thus far, clinical studies of antiviral hepatitis C agents have not specifically addressed whether the participating individuals had exhibited virally-induced photosensitivity prior to receiving the treatment [1-7,10-20].

## Conclusions

Antiviral therapy for hepatitis C (individual and combination drugs) may lead to cutaneous adverse events, including photosensitivity. We reported the case of a man with chronic hepatitis C virus infection who was successfully treated with sofosbuvir-velpatasvir. He developed a prominent drug-related phototoxic eruption that presented shortly after initiating the drug. The phototoxic skin reaction completely resolved once he completed treatment. Once the diagnosis of medication-induced phototoxicity was established, he began interventions to prevent sun exposure to his skin. The incidence of anti-hepatitis C viral agents-induced phototoxicity is variable and depends on the specific drug that is being used for treatment. However, clinicians should consider counseling the patients to use adequate topical sunscreen and photoprotective clothing to prevent exposure of their skin to the sun.
